# Function of the Pharmacist

**Published:** 1893-08

**Authors:** 


### FUNCTION OF THE PHARMACIST.

The JOURNAL had always thought that the relation borne by the great manufacturing pharmacist to the medical profession was that of a caterer, that he was the doctor's ally, in that he materialized the discoveries made by the physician with regard to therapeutics, and prepared the medicines needed in practice, ready to his hand; that he had taken, on a large scale, the place of the "apothecary" or compounder of drugs for the doctor, of old ancient times,—relieving the doctor of all the details of the mere preparation for use. Great improvement has been made in respect to the form in which medicines are now dispensed. In concentrated form, neat and attractive in appearance, and stripped of all repulsiveness in taste or smell, all the medicines needed by the practitioner can now be carried in a buggy case or pocket case.

Parke, Davis & Co. have for years been recognized as pioneers in this line. Anticipating the demand for some drug, which, through the recommendation of some prominent practitioner (who has given the profession the benefit of his experience) has become popular,—or, in modern parlance—the "fad," this firm has usually been the first to put it upon the market in eligible form and of definite and reliable strength, and to advertise it to the profession in a strictly legitimate way. Other large firms have been alongside of them,—Sharp & Dohme, Wyeth Bros., W. H. Scheffelin and others,—and they serve the profession very acceptably by their tablets, triturates, fluid extracts, etc.

The medical profession have shown a keen appreciation of this enterprise by the bestowal of a liberal patronage. These great manufacturing pharmacists do not hesitate to take hold of any new drug which comes to the surface, and investigate it thoroughly. They expend large sums of money, both for the crude drug and for machinery and apparatus with which to analyze it, and if found valuable they prepare it in elegant form ready for administration. They extract the active principle, most frequently an alkaloid, and enable the doctor to administer it hypodermically.

Looking at the relation of the two professions in this light, and having for many years observed the intelligent zeal with which these big firms have followed up the suggestions of the leaders of medicine by supplying the profession with any drug discovered, or combination recommended, we were no little surprised to see the spirit of indignation with which Dr. Hammond protested against the manufacture and sale by Parke, Davis & Co. of "certain animal extracts." It has led to an unfortunate and bitter controversy, from which can come no good to any one, that we see.

It will be remembered that Dr. Hammond read a paper before the N. Y. Post Graduate Faculty and class on "Certain Animal Extracts" in the treatment of certain diseases, and detailed quite a lengthy experience he had had with a preparation which he called "cerebrine," made from an ox brain by his own hand. This paper was ostensibly a scientific paper, and read for the benefit of the profession. It was published in the *New York Medical Journal* and extensively copied.

What was more natural then, and to be expected, than that leading manufacturing pharmacists should, on so strong recommendation from such well known authority, anticipate that there would be a demand for these extracts, and that they should hasten to prepare them and offer them for sale? Parke, Davis & Co. purchased machinery and apparatus and begun the manufacture of several extracts "in accordance with Dr. Hammond's recommendation," amongst others, one from ox brain, which they called "cerebrin,"—Dr. Hammond having called that prepared by himself "cerebrine." Dr. Hammond stated that it required six months preparation. Parke, Davis & Co. announced that by a *process of their own*, they were enabled to prepare "cerebrin" in six days, and claimed that it had the same virtue and strength as if prepared in six months. Dr. Hammond demanded that they should cease using his name and cease making cerebrin—as it was *an infringement upon his rights!* Parke, Davis & Co. then at once called in the advertisements they had sent out and we presumed the trouble was over.

Since then, however, a very fiery correspondence has taken place between the parties, or rather, between the lawyers of the parties, and the medical profession are threatened to be deluged with the details of another unnecessary and vexatious lawsuit, a quarrel between a doctor and a manufacturer of drugs. The latter have sent out a circular letter to the medical press, from which, in the interest of fair play, we copy the following.

They claim that they "had no means of knowing that the article in the *New York Medical Journal* was *intended as an advertisement* for a manufacturing house, or that the extracts advocated therein were *protected* in any form by trade-mark." And again, they say:

"If Dr. Hammond had stated in his paper that . . . he was President of the . . . Chemical Company, and that he claimed proprietorship in the words 'Cerebrine,' etc., and that the extracts advocated in the paper were manufactured in his own laboratory . . . solely for the . . . Chemical Company, is it not probable that his paper would have been rejected by the *New York Medical Journal?*

"On the contrary, this paper appeared in the guise of a scientific communication, and as such, was accepted by the medical press generally, and given wide dissemination. We too were deceived among the rest, and consequently, in our desire to keep abreast of the times in the introduction of new therapeutic agents, we were led to manufacture, advertise and market the alleged principles recommended by Dr. Hammond. For this act we are threatened, through the attorneys of Dr. Hammond, with dire penalties. And not content with threatened legal punishment, Dr. Hammond disseminates through the mails a scurrilous circular that indicates intent to do great injustice to us, and finally enlists the medical press (*New England Medical Monthly*, Dr. Wile) in circulating this personal attack."

As the TEXAS MEDICAL JOURNAL declined to publish the article referred to, though several times requested by an advertising firm to do so, typewritten copies of the original manuscript being sent us in advance of publication elsewhere, and as we have so far taken no part in the controversy,—certainly have not published Dr. Hammond's circular, we object to the above remark that "the *medical press*" had been "enlisted" in circulating a "scurrilous attack." But we echo the sentiment expressed by Parke, Davis & Co., that it is time to pause and ask whither away medicine is drifting and what of the code of ethics, if the medical journals are to be used for such purpose without protest.

The JOURNAL would remain neutral in this matter, as both parties are patrons and friends; but this is not a personal matter. There is principle involved, and if leading practitioners of medicine are to become proprietors of trade-marked preparations of of their own discoveries, we see no distinction whatever between cases of this kind and that of Amick, and of Keely, against whom such an outcry has been made, and we had as well now as later, break down all barriers, throw the code to the winds,—"let slip the dogs of war and cry havoc," and every one go in on the make,—for with the *aid of the medical press* it would seem to be rapidly coming to that. I am afraid Wile has gone back on his record,—he used to speak out in defense of legitimate medicine.

